# 357. A Comparison of Chest CT Findings in Cancer and Non-Cancer Patients with COVID-19

**DOI:** 10.1093/ofid/ofab466.558

**Published:** 2021-12-04

**Authors:** Alexandre Malek, Hiba Dagher, Ray Y Hachem, Ying Jiang, Anne-Marie Chaftari, Issam I Raad

**Affiliations:** 1 UT MD Anderson Cancer Center, Houston, TX; 2 MD Anderson Cancer Center, Houston, TX; 3 The University of Texas MD Anderson Cancer Center, Houston, TX

## Abstract

**Background:**

The purpose of this study was to compare chest computed tomography (CT) scan findings in cancer versus non-cancer patients with COVID-19 infection. We sought to assess the correlation between radiologic patterns of COVID-19 pneumonia, clinical course, and outcomes.

**Methods:**

We performed a retrospective study of COVID-19 positive cancer and non-cancer pts who had chest CT scans at the time of diagnosis, at our hospital and 16 other centers in Asia, Australia, Europe, North America and South America, between March, 2020 and November, 2020. Patients’ age, underlying diseases, symptoms, laboratory studies, and radiologic findings consisting of bilateral ground-glass opacities (GGOs), multifocal organizing pneumonia (MOP) were collected in association with clinical outcomes.

**Results:**

We identified 426 pts with cancer and 622 non-cancer pts. Thereafter, cancer pts were analyzed into 3 distinct groups and similar to non-cancer pts: GGOs group (n=224, 54%), GGOs+MOP group (n=61, 14.6%), and a third group of neither GGOs or MOP (n=131, 31.4%) in cancer pts, and in non-cancer pts: GGOs group (n=387, 62.8%), GGOs +MOP group (n=100, 16.2%), and a third group of neither GGOs or MOP (n=129, 21%). The median patients’ age was 54 in non-cancer pts vs 62 in cancer pts (p< 0.001) and there were more males in the non-cancer group 57% vs 47% (p=0.001). Cough was more prevalent in non-cancer pts, 71% vs 59% (p< 0.001) and similar to fever (73% vs 57%, p< 0.001). Neutropenia < 0.5 k/µL and lymphocytopenia < 1 k/µL were more frequent in cancer pts (p< 0.001). In cancer pts, there was no statistically significance difference between the 3 groups (hospital admission, mechanical ventilation, readmission within 30 days, and mortality), except pts who required non-invasive (NI) ventilation were more frequent in the GGOs group, 55% (p=0.005). In non-cancer, pts with GGOs +MOP have higher hospital admission, ICU transfer, NI- and mechanical ventilation compared to the 2 other groups (p< 0.001). While readmission to hospital or mortality rate within 30 days were similar between the 3 groups.

**Conclusion:**

This study reveals that non-cancer pts tended to have more radiologic findings on chest CT scan compared to cancer pts at the time of COVID-19 diagnosis and were associated with more worrisome COVID-19-related clinical outcomes.

**Disclosures:**

**All Authors**: No reported disclosures

